# *N*-(2,5-Dichloro­phen­yl)benzene­sulfonamide

**DOI:** 10.1107/S1600536810004769

**Published:** 2010-02-13

**Authors:** B. Thimme Gowda, Sabine Foro, P. G. Nirmala, Hartmut Fuess

**Affiliations:** a Department of Chemistry, Mangalore University, Mangalagangotri 574199, Mangalore, India; b Institute of Materials Science, Darmstadt University of Technology, Petersenstrasse 23, D-64287, Darmstadt, Germany

## Abstract

In the title compound, C_12_H_9_Cl_2_NO_2_S, the conformation of the N—H bond is *syn* to the 2-chloro group and *anti* to the 3-chloro group of the aniline benzene ring. The mol­ecule is bent at the *S* atom with a C—SO_2_—NH—C torsion angle of 66.4 (2)°. The two rings form a dihedral angle of 73.3 (1)° and an intra­molecular N—H⋯Cl hydrogen bond occurs. The crystal structure features chains linked by N—H⋯O hydrogen bonds.

## Related literature

For the preparation of the title compound, see: Shetty & Gowda (2005 ▶). For our study of the effect of substituents on the structures of *N*-(ar­yl)aryl­sulfonamides, see: Gowda *et al.* (2009 ▶, 2010 ▶). For related structures, see: Gelbrich *et al.* (2007 ▶); Perlovich *et al.* (2006 ▶).
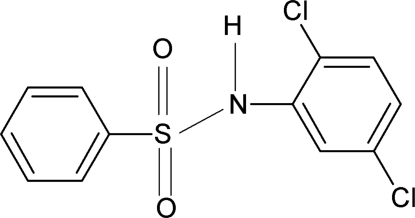


## Experimental

### 

#### Crystal data

C_12_H_9_Cl_2_NO_2_S
*M* *_r_* = 302.16
Monoclinic, 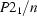

*a* = 9.595 (1) Å
*b* = 14.188 (2) Å
*c* = 10.424 (1) Å
β = 114.42 (2)°
*V* = 1292.1 (3) Å^3^
*Z* = 4
Mo *K*α radiation
μ = 0.66 mm^−1^
*T* = 299 K
0.44 × 0.40 × 0.32 mm

#### Data collection

Oxford Diffraction Xcalibur diffractometer with a Sapphire CCD Detector
Absorption correction: multi-scan (*CrysAlis RED*; Oxford Diffraction, 2009 ▶) *T*_min_ = 0.761, *T*_max_ = 0.818
5199 measured reflections
2638 independent reflections
2225 reflections with *I* > 2σ(*I*)
*R*_int_ = 0.010

#### Refinement

*R*[*F* ^2^ > 2σ(*F* ^2^)] = 0.031
*wR*(*F* ^2^) = 0.086
*S* = 1.05
2638 reflections
167 parameters
1 restraint
H atoms treated by a mixture of independent and constrained refinement
Δρ_max_ = 0.23 e Å^−3^
Δρ_min_ = −0.34 e Å^−3^

### 

Data collection: *CrysAlis CCD* (Oxford Diffraction, 2009 ▶); cell refinement: *CrysAlis RED* (Oxford Diffraction, 2009 ▶); data reduction: *CrysAlis RED*; program(s) used to solve structure: *SHELXS97* (Sheldrick, 2008 ▶); program(s) used to refine structure: *SHELXL97* (Sheldrick, 2008 ▶); molecular graphics: *PLATON* (Spek, 2009 ▶); software used to prepare material for publication: *SHELXL97*.

## Supplementary Material

Crystal structure: contains datablocks I, global. DOI: 10.1107/S1600536810004769/fl2289sup1.cif

Structure factors: contains datablocks I. DOI: 10.1107/S1600536810004769/fl2289Isup2.hkl

Additional supplementary materials:  crystallographic information; 3D view; checkCIF report

## Figures and Tables

**Table 1:** Hydrogen-bond geometry (Å, °)

*D*—H⋯*A*	*D*—H	H⋯*A*	*D*⋯*A*	*D*—H⋯*A*
N1—H1*N*⋯O1^i^	0.84 (1)	2.28 (1)	3.074 (2)	156 (2)
N1—H1*N*⋯Cl1	0.84 (1)	2.52 (2)	2.9795 (16)	115 (2)

Symmetry code: (i) 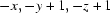.

**Fig. 1.**
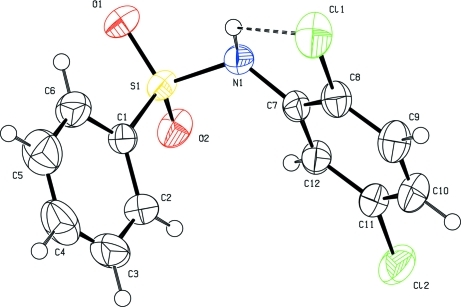
Molecular structure of (I), showing the atom labelling and displacement ellipsoids drawn at the 50% probability level.

**Fig. 2.**
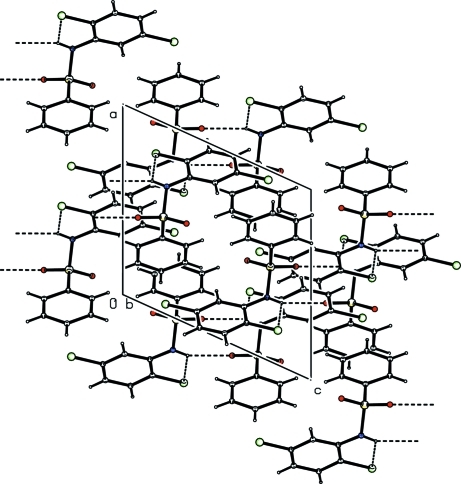
Molecular packing of (I) with hydrogen bonding shown as dashed lines.

## supplementary crystallographic information

### Comment

As part of a study of substituent effects on the structures of *N*-(aryl)arylsulfonamides (Gowda *et al.*, 2009; 2010), the structure of (I) has been determined. The conformation of the N—H bond is *syn* to the 2-chloro group and *anti* to the 3-chloro group in the aniline benzene ring (Fig. 1). The molecule is bent at the *S* atom with the C—SO_2_—NH—C torsion angle of 66.4 (2)°, compared to the values of -62.1 (3)° and 60.7 (3)°, in the two molecules of *N*-(2,4-dichlorophenyl)benzenesulfonamide (II), -68.1 (3)° in *N*-(3,5-dichlorophenyl)benzenesulfonamide (III) (Gowda *et al.*, 2010) and 62.7 (2)° in *N*-(2,5-dimethylphenyl)benzenesulfonamide (IV) (Gowda *et al.*, 2009).

The sulfonyl benzene and the aniline benzene rings in (I) are tilted relative to each other by 73.3 (1)°, compared to the values of 70.8 (1)° (molecule 1) and 74.8 (1)° (molecule 2) in (II), 57.0 (1)° in (III) and 40.4 (1)° in (IV). The other bond parameters in (I) are similar to those observed in (II)-(IV) and other aryl sulfonamides (Perlovich *et al.*, 2006; Gelbrich *et al.*, 2007).

An intramolecular N—H···Cl hydrogen bond is observed. The crystal packing of molecules in (I) is *via* N—H···O(S) hydrogen bonds (Table 1, Fig. 2).

### Experimental

The solution of benzene (10 ml) in chloroform (40 ml) was treated dropwise with chlorosulfonic acid (25 ml) at 0 ° C. After the initial evolution of hydrogen chloride subsided, the reaction mixture was brought to room temperature and poured into crushed ice in a beaker. The chloroform layer was separated, washed with cold water and allowed to evaporate slowly. The residual benzenesulfonylchloride was treated with 2,5-dichloroaniline in the stoichiometric amounts and boiled for ten minutes. The reaction mixture was then cooled to room temperature and added to ice cold water (100 ml). The resultant solid (I) was filtered under suction and washed thoroughly with cold water. It was then recrystallized to constant melting point from dilute ethanol. The purity of the compound was checked and characterized by recording its infrared and NMR spectra (Shetty & Gowda, 2005). The rod like colorless single crystals used in X-ray diffraction studies were grown in ethanolic solution by evaporating it at room temperature.

### Refinement

The H atom of the NH group was located in a difference map and later restrained to the distance N—H = 0.86 (1) Å. The other H atoms were positioned with idealized geometry using a riding model with C—H = 0.93 Å A l l H atoms were refined with isotropic displacement parameters (set to 1.2 times of the *U*_eq_ of the parent atom).

### Crystal data

**Table:**

C_12_H_9_Cl_2_NO_2_S	*F*(000) = 616
*M**_r_* = 302.16	*D*_x_ = 1.553 Mg m^−^^3^
Monoclinic, *P*2_1_/*n*	Mo *K*α radiation, λ = 0.71073 Å
Hall symbol: -P 2yn	Cell parameters from 2765 reflections
*a* = 9.595 (1) Å	θ = 2.7–27.8°
*b* = 14.188 (2) Å	µ = 0.66 mm^−^^1^
*c* = 10.424 (1) Å	*T* = 299 K
β = 114.42 (2)°	Prism, colourless
*V* = 1292.1 (3) Å^3^	0.44 × 0.40 × 0.32 mm
*Z* = 4	

### Data collection

**Table:**

Oxford Diffraction Xcalibur diffractometer with a Sapphire CCD Detector	2638 independent reflections
Radiation source: fine-focus sealed tube	2225 reflections with *I* > 2σ(*I*)
graphite	*R*_int_ = 0.010
Rotation method data acquisition using ω and phi scans.	θ_max_ = 26.4°, θ_min_ = 2.7°
Absorption correction: multi-scan (*CrysAlis RED*; Oxford Diffraction, 2009)	*h* = −11→11
*T*_min_ = 0.761, *T*_max_ = 0.818	*k* = −13→17
5199 measured reflections	*l* = −7→13

### Refinement

**Table:**

Refinement on *F*^2^	Secondary atom site location: difference Fourier map
Least-squares matrix: full	Hydrogen site location: inferred from neighbouring sites
*R*[*F*^2^ > 2σ(*F*^2^)] = 0.031	H atoms treated by a mixture of independent and constrained refinement
*wR*(*F*^2^) = 0.086	*w* = 1/[σ^2^(*F*_o_^2^) + (0.0435*P*)^2^ + 0.4815*P*] where *P* = (*F*_o_^2^ + 2*F*_c_^2^)/3
*S* = 1.05	(Δ/σ)_max_ = 0.001
2638 reflections	Δρ_max_ = 0.23 e Å^−^^3^
167 parameters	Δρ_min_ = −0.34 e Å^−^^3^
1 restraint	Extinction correction: *SHELXL*, Fc^*^=kFc[1+0.001xFc^2^λ^3^/sin(2θ)]^-1/4^
Primary atom site location: structure-invariant direct methods	Extinction coefficient: 0.0316 (18)

### Special details

**Table:**

Experimental. Empirical absorption correction using spherical harmonics, implemented in SCALE3 ABSPACK scaling algorithm.
Geometry. All esds (except the esd in the dihedral angle between two l.s. planes) are estimated using the full covariance matrix. The cell esds are taken into account individually in the estimation of esds in distances, angles and torsion angles; correlations between esds in cell parameters are only used when they are defined by crystal symmetry. An approximate (isotropic) treatment of cell esds is used for estimating esds involving l.s. planes.
Refinement. Refinement of *F*^2^ against ALL reflections. The weighted *R*-factor wR and goodness of fit *S* are based on *F*^2^, conventional *R*-factors *R* are based on *F*, with *F* set to zero for negative *F*^2^. The threshold expression of *F*^2^ > σ(*F*^2^) is used only for calculating *R*-factors(gt) etc. and is not relevant to the choice of reflections for refinement. *R*-factors based on *F*^2^ are statistically about twice as large as those based on *F*, and *R*- factors based on ALL data will be even larger.

### Fractional atomic coordinates and isotropic or equivalent isotropic displacement parameters (Å^2^)

**Table:**

	*x*	*y*	*z*	*U*_iso_*/*U*_eq_	
C1	−0.10881 (19)	0.57873 (12)	0.71058 (18)	0.0342 (4)	
C2	−0.0940 (2)	0.61982 (15)	0.8359 (2)	0.0482 (5)	
H2	−0.0255	0.5957	0.9217	0.058*	
C3	−0.1826 (3)	0.69707 (18)	0.8311 (3)	0.0665 (7)	
H3	−0.1732	0.7259	0.9145	0.080*	
C4	−0.2852 (3)	0.73206 (17)	0.7038 (3)	0.0690 (7)	
H4	−0.3452	0.7840	0.7016	0.083*	
C5	−0.2991 (3)	0.69043 (18)	0.5800 (3)	0.0675 (7)	
H5	−0.3688	0.7143	0.4943	0.081*	
C6	−0.2105 (2)	0.61368 (15)	0.5821 (2)	0.0501 (5)	
H6	−0.2188	0.5858	0.4984	0.060*	
C7	0.27503 (19)	0.56534 (12)	0.85564 (18)	0.0338 (4)	
C8	0.3535 (2)	0.64497 (13)	0.8424 (2)	0.0403 (4)	
C9	0.4606 (2)	0.68914 (15)	0.9591 (2)	0.0515 (5)	
H9	0.5129	0.7414	0.9479	0.062*	
C10	0.4906 (2)	0.65644 (16)	1.0922 (2)	0.0528 (5)	
H10	0.5621	0.6865	1.1713	0.063*	
C11	0.4128 (2)	0.57828 (14)	1.10589 (19)	0.0429 (4)	
C12	0.3074 (2)	0.53198 (13)	0.99013 (18)	0.0386 (4)	
H12	0.2581	0.4785	1.0022	0.046*	
N1	0.17095 (17)	0.51827 (11)	0.73345 (15)	0.0363 (3)	
H1N	0.166 (2)	0.5403 (13)	0.6564 (14)	0.044*	
O1	−0.05974 (16)	0.43413 (9)	0.58100 (13)	0.0443 (3)	
O2	0.02450 (15)	0.42616 (9)	0.83833 (13)	0.0462 (3)	
Cl1	0.31855 (6)	0.68832 (4)	0.67634 (6)	0.05849 (18)	
Cl2	0.44864 (7)	0.53545 (5)	1.27273 (5)	0.06258 (19)	
S1	0.00256 (5)	0.47941 (3)	0.71548 (4)	0.03304 (14)	

### Atomic displacement parameters (Å^2^)

**Table:**

	*U*^11^	*U*^22^	*U*^33^	*U*^12^	*U*^13^	*U*^23^
C1	0.0319 (9)	0.0366 (9)	0.0350 (9)	−0.0051 (7)	0.0147 (7)	−0.0037 (7)
C2	0.0452 (11)	0.0609 (13)	0.0406 (10)	−0.0044 (9)	0.0200 (9)	−0.0115 (9)
C3	0.0580 (14)	0.0735 (16)	0.0783 (17)	−0.0084 (12)	0.0385 (13)	−0.0330 (14)
C4	0.0510 (13)	0.0540 (14)	0.109 (2)	0.0053 (11)	0.0400 (14)	−0.0137 (14)
C5	0.0563 (14)	0.0647 (15)	0.0737 (16)	0.0185 (12)	0.0190 (12)	0.0078 (13)
C6	0.0473 (11)	0.0551 (12)	0.0418 (11)	0.0082 (9)	0.0125 (9)	−0.0003 (9)
C7	0.0272 (8)	0.0366 (9)	0.0352 (9)	0.0026 (7)	0.0106 (7)	−0.0005 (7)
C8	0.0344 (9)	0.0397 (10)	0.0434 (10)	0.0020 (7)	0.0126 (8)	0.0064 (8)
C9	0.0441 (11)	0.0457 (11)	0.0587 (13)	−0.0129 (9)	0.0152 (9)	−0.0018 (9)
C10	0.0427 (11)	0.0575 (13)	0.0477 (11)	−0.0115 (9)	0.0083 (9)	−0.0106 (10)
C11	0.0342 (9)	0.0540 (12)	0.0351 (9)	0.0011 (8)	0.0090 (7)	−0.0019 (8)
C12	0.0333 (9)	0.0421 (10)	0.0376 (9)	−0.0028 (7)	0.0118 (8)	0.0004 (8)
N1	0.0340 (8)	0.0450 (9)	0.0306 (7)	−0.0030 (6)	0.0140 (6)	−0.0007 (6)
O1	0.0510 (8)	0.0428 (7)	0.0338 (7)	−0.0064 (6)	0.0123 (6)	−0.0099 (5)
O2	0.0507 (8)	0.0454 (7)	0.0357 (7)	−0.0107 (6)	0.0112 (6)	0.0078 (6)
Cl1	0.0530 (3)	0.0621 (3)	0.0535 (3)	−0.0064 (2)	0.0152 (2)	0.0205 (3)
Cl2	0.0564 (3)	0.0871 (4)	0.0342 (3)	−0.0090 (3)	0.0085 (2)	0.0007 (3)
S1	0.0356 (2)	0.0331 (2)	0.0272 (2)	−0.00569 (17)	0.00974 (17)	−0.00172 (16)

### Geometric parameters (Å, °)

**Table:**

C1—C6	1.383 (3)	C7—N1	1.419 (2)
C1—C2	1.383 (2)	C8—C9	1.377 (3)
C1—S1	1.7565 (18)	C8—Cl1	1.7343 (19)
C2—C3	1.375 (3)	C9—C10	1.375 (3)
C2—H2	0.9300	C9—H9	0.9300
C3—C4	1.377 (4)	C10—C11	1.377 (3)
C3—H3	0.9300	C10—H10	0.9300
C4—C5	1.375 (4)	C11—C12	1.379 (2)
C4—H4	0.9300	C11—Cl2	1.737 (2)
C5—C6	1.376 (3)	C12—H12	0.9300
C5—H5	0.9300	N1—S1	1.6430 (15)
C6—H6	0.9300	N1—H1N	0.844 (9)
C7—C12	1.388 (2)	O1—S1	1.4292 (13)
C7—C8	1.396 (2)	O2—S1	1.4254 (13)
			
C6—C1—C2	121.31 (18)	C7—C8—Cl1	119.74 (14)
C6—C1—S1	119.54 (14)	C10—C9—C8	120.44 (19)
C2—C1—S1	119.15 (15)	C10—C9—H9	119.8
C3—C2—C1	118.8 (2)	C8—C9—H9	119.8
C3—C2—H2	120.6	C9—C10—C11	118.67 (18)
C1—C2—H2	120.6	C9—C10—H10	120.7
C2—C3—C4	120.4 (2)	C11—C10—H10	120.7
C2—C3—H3	119.8	C10—C11—C12	121.72 (18)
C4—C3—H3	119.8	C10—C11—Cl2	119.63 (15)
C5—C4—C3	120.2 (2)	C12—C11—Cl2	118.65 (15)
C5—C4—H4	119.9	C11—C12—C7	119.90 (17)
C3—C4—H4	119.9	C11—C12—H12	120.1
C4—C5—C6	120.4 (2)	C7—C12—H12	120.1
C4—C5—H5	119.8	C7—N1—S1	123.65 (12)
C6—C5—H5	119.8	C7—N1—H1N	115.0 (15)
C5—C6—C1	118.9 (2)	S1—N1—H1N	110.3 (15)
C5—C6—H6	120.6	O2—S1—O1	119.31 (8)
C1—C6—H6	120.6	O2—S1—N1	107.60 (8)
C12—C7—C8	118.11 (16)	O1—S1—N1	104.80 (8)
C12—C7—N1	121.79 (16)	O2—S1—C1	108.27 (8)
C8—C7—N1	120.03 (15)	O1—S1—C1	109.23 (8)
C9—C8—C7	121.14 (18)	N1—S1—C1	106.97 (8)
C9—C8—Cl1	119.11 (15)		
			
C6—C1—C2—C3	−0.1 (3)	C9—C10—C11—Cl2	179.98 (17)
S1—C1—C2—C3	−179.45 (16)	C10—C11—C12—C7	−1.5 (3)
C1—C2—C3—C4	0.7 (3)	Cl2—C11—C12—C7	179.16 (13)
C2—C3—C4—C5	−0.5 (4)	C8—C7—C12—C11	1.0 (3)
C3—C4—C5—C6	−0.2 (4)	N1—C7—C12—C11	178.02 (17)
C4—C5—C6—C1	0.7 (4)	C12—C7—N1—S1	45.9 (2)
C2—C1—C6—C5	−0.5 (3)	C8—C7—N1—S1	−137.14 (15)
S1—C1—C6—C5	178.75 (18)	C7—N1—S1—O2	−49.78 (17)
C12—C7—C8—C9	0.3 (3)	C7—N1—S1—O1	−177.73 (14)
N1—C7—C8—C9	−176.78 (18)	C7—N1—S1—C1	66.36 (16)
C12—C7—C8—Cl1	179.19 (13)	C6—C1—S1—O2	−146.50 (15)
N1—C7—C8—Cl1	2.1 (2)	C2—C1—S1—O2	32.81 (16)
C7—C8—C9—C10	−1.2 (3)	C6—C1—S1—O1	−15.12 (18)
Cl1—C8—C9—C10	179.95 (17)	C2—C1—S1—O1	164.19 (14)
C8—C9—C10—C11	0.7 (3)	C6—C1—S1—N1	97.79 (16)
C9—C10—C11—C12	0.6 (3)	C2—C1—S1—N1	−82.90 (16)

### Hydrogen-bond geometry (Å, °)

**Table:**

*D*—H···*A*	*D*—H	H···*A*	*D*···*A*	*D*—H···*A*
N1—H1N···O1^i^	0.84 (1)	2.28 (1)	3.074 (2)	156 (2)
N1—H1N···Cl1	0.84 (1)	2.52 (2)	2.9795 (16)	115 (2)

Symmetry codes: (i) −*x*, −*y*+1, −*z*+1.
